# Influence of Polydatin on the Tumor Microenvironment In Vitro: Studies with a Colon Cancer Cell Model

**DOI:** 10.3390/ijms23158442

**Published:** 2022-07-29

**Authors:** Alex De Gregorio, Ewa Krystyna Krasnowska, Manuela Zonfrillo, Giampietro Ravagnan, Valentina Bordignon, Enzo Bonmassar, Maria Pia Fuggetta

**Affiliations:** 1 Institute of Translational Pharmacology, National Council of Research (CNR), Via Fosso del Cavaliere, 00133 Rome, Italy; alex.degregorio@ift.cnr.it (A.D.G.); ewa.krasnowska@ift.cnr.it (E.K.K.); manuela.zonfrillo@ift.cnr.it (M.Z.); ravagnangp@gmail.com (G.R.); bonmasse@yahoo.com (E.B.); 2 Life and Earth Science Faculty, Selinus University of Sciences and Literature, 71-75 Shelton Street, London WC2H 9JQ, UK; valentinabordignon@hotmail.com

**Keywords:** polydatin, colorectal cancer (CRC), inflammation, tumor microenvironment (TME), cytokine, apoptosis, cellular adhesion molecules (CAMs), coculture, integrative oncology

## Abstract

The tumor microenvironment of colon carcinoma, the site at which tumor cells and the host immune system interact, is influenced by signals from tumor cells, immunocompetent cells, and bacterial components, including LPS. A large amount of LPS is available in the colon, and this could promote inflammation and metastasis by enhancing tumor cell adhesion to the endothelium. Polydatin (PD), the 3-β-D-glucoside of trans-resveratrol, is a polyphenol with anti-cancer, anti-inflammatory, and immunoregulatory effects. This study was designed to explore whether PD is able to produce antiproliferative effects on three colon cancer lines, to reduce the expression of adhesion molecules that are upregulated by LPS on endothelial cells, and to decrease the proinflammatory cytokines released in culture supernatants. Actually, we investigated the effects of PD on tumor growth in a coculture model with human mononuclear cells (MNCs) that mimics, at least in part, an in vitro tumor microenvironment. The results showed that PD alone or in combination with MNC exerts antiproliferative and proapoptotic effects on cancer cells, inhibits the production of the immunosuppressive cytokine IL-10 and of the proinflammatory cytokines upregulated by LPS, and reduces E-selectin and VCAM-1 on endothelial cells. These data provide preclinical support to the hypothesis that PD could be of potential benefit as a therapeutic adjuvant in colon cancer treatment and prevention.

## 1. Introduction

Colorectal cancer (CRC) is listed among the most common neoplastic diseases worldwide. The incidence of CRC increases with aging and depends, at least in part, on genetic patterns, lifestyle, and environmental factors [1]. The prognosis is determined by the extent of local growth and mainly by the metastatic spreading that is the major cause of cancer-related death. In patients with advanced metastatic CRC (i.e., stage 4 CRC), metastases are clinically detectable mainly in the lungs and liver, with a 5-year survival rate of less than 10% [2].

Tumor growth and metastasis are determined not only by genetic and epigenetic alterations inside the cell, such as oncogene activation and proliferative signals, but also by an inflamed tumor microenvironment (TME) that plays an essential role in the progression of the disease. Several components of the TME contribute to metastatic progression, including angiogenesis, epithelial–mesenchymal transition, stromal cell support, immunocyte/inflammatory cell infiltration, cytokine/chemokine disorders, and cell–cell interactions. In the TME, the inflammation is driven by proinflammatory mediators that are secreted, following the cross -talk among tumor, stromal, and immune cells. Hence, the production of mediators of inflammation and cell-mediated immunosuppression are supposed to favor disease progression, i.e., tumor invasion, metastasis, and neo-angiogenesis [3,4,5,6,7].

Among the inflammatory cells recruited to the tumor site, macrophages (i.e., tumor-associated macrophages, TAMs) play a crucial role in CRC [6] by secreting cytokines and chemokines with proinflammatory and immunosuppressive effects [7]. In particular, interleukin-10 (IL-10), derived from TAMs, induces the accumulation of regulatory T cells (Tregs) that contribute to tumor aggressiveness [8,9,10].

CRC cells are in contact with bacteria that produce copious amounts of lipopolysaccharide (LPS) that can cause systemic inflammation by interacting with host immune and inflammatory mediator cells [11] and promotes colon cancer metastasis by enhancing inflammation and tumor cell adhesion, intravasation, and extravasation [12].

The effects of LPS are mediated through its interaction with several receptors for microbial products comprising Toll-like receptors (TLRs) that are a group of type 1 transmembrane proteins able to recognize a variety of molecular signals, including endogenous-damage- and pathogen-associated signals. The best-studied member of the TLR family is TLR-4, involved in the recognition of endotoxins or bacterial LPS [13,14,15].

LPS-induced TLR-4 signaling leads to the activation of various downstream mitogen-activated protein kinases (MAPKs) that have been shown to play key roles in cell proliferation, apoptosis, cell adhesion [16], and production of various proinflammatory and microbicidal molecules [17].

One of the initial steps in the metastatic process concerns the adhesion of malignant cells to the vascular endothelium. Adhesion is made possible by the expression of cellular adhesion molecules (CAMs) on tumor cells [18,19] and by E-selectin [20] present on the endothelium of tumor vessels. Notably, E-selectin is minimally expressed on the resting endothelium and is transcriptionally induced by cytokines and by bacterial LPS [21,22], thus favoring metastasis generation [23].

Polydatin (3,5,4-trihydroxystilbene 3-O-beta-D-glucopyranosid, PD) is a glycoside of resveratrol (RES) in which the glycoside group is bound to the C-3 position and substitutes a hydroxyl group. PD is more efficiently absorbed by the oral route and is more resistant to enzymatic oxidation than RES. In contrast to RES, which penetrates cells passively, PD enters cells via an active mechanism based on glucose carriers. These properties provide PD with greater bioavailability compared to RES [24,25].

Previous studies performed in our laboratory have given noticeable support to the observation that RES possesses potent anticancer and immunomodulant activity [26,27]. Moreover, PD is known to have various beneficial effects not only as an anti-cancer natural compound but also as an antioxidant, anti-inflammatory, and immune-modulating agent [27,28,29,30,31,32].

The aim of this study was to investigate in vitro the capability of PD to control the growth of human colon tumor cells in terms of proliferation inhibition and apoptosis induction. Moreover, we adopted an in vitro simplified model of the inflamed CRC microenvironment using LPS as a proinflammatory stimulus to investigate the effects of PD on the production of proinflammatory mediators, on the production of IL-10, and on endothelial CAM proteins.

## 2. Results

### 2.1. Antiproliferative Effect of PD on CRC and CRC/MNC Coculture

**Figure 1:**
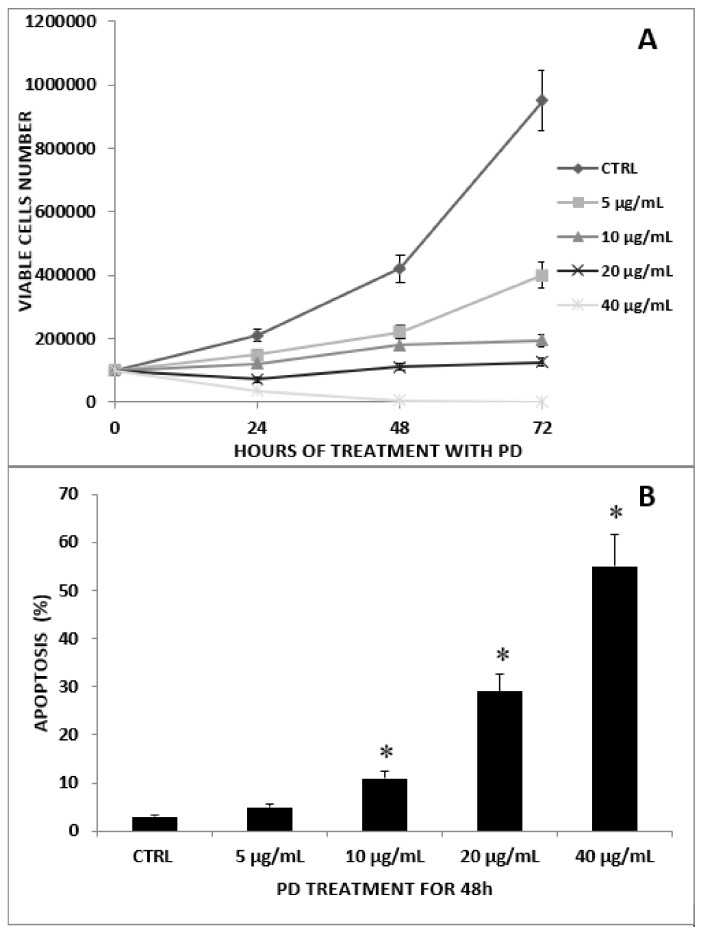
HT-29 cell growth and apoptosis induction. (**A**) Time course of the antiproliferative effect of graded concentrations of PD on the HT-29 tumor cell line. The cell proliferation was determined by cell counts in trypan blue. Data are expressed in terms of the number of viable cells. Measurements were done in triplicate, and bars represent ±SE of the mean of three separate counts. Values are referred to a representative experiment from at least three independent experiments. (**B**) Apoptosis induction in HT-29 cancer cells with graded concentrations of PD. Cells were cultured in CM alone (control) or CM containing the indicated concentrations of PD for 48 h. At the end of the culture period, the cells were harvested and the percentages of apoptotic cells were evaluated by flow cytometry. Data expressed as the mean of the percentage of cells in apoptosis from at least three independent experiments. * *p* ≤ 0.01 with respect to untreated controls.

The kinetics of HT-29 cell growth after exposure to graded concentrations of PD (5, 10, 20, and 40 µg/mL,) for 24, 48, and 72 h is illustrated in Figure 1A.

The results show that PD significantly reduced the exponential growth of tumor cells in a concentration- and time-dependent manner. At 40 μg/mL, the drug showed a potent cytotoxic effect. According to dye exclusion assay, more than 90% of treated tumor cells were found to be dead after 3-day treatment. After 48 h of culture, significant growth inhibition, reaching approximately 50%, was observed using lower PD concentrations (e.g., 5 µg/mL).

To explore further the modality of PD-induced cytotoxicity, we analyzed the extent of HT-29 cell apoptosis following exposure to the drug for 48 h using flow cytometric analysis. The results (Figure 1B) show that PD induced apoptosis in a concentration/effect manner that reached a statistically significant level starting from 10 µg/mL (*p* < 0.01) onward.

**Figure 2:**
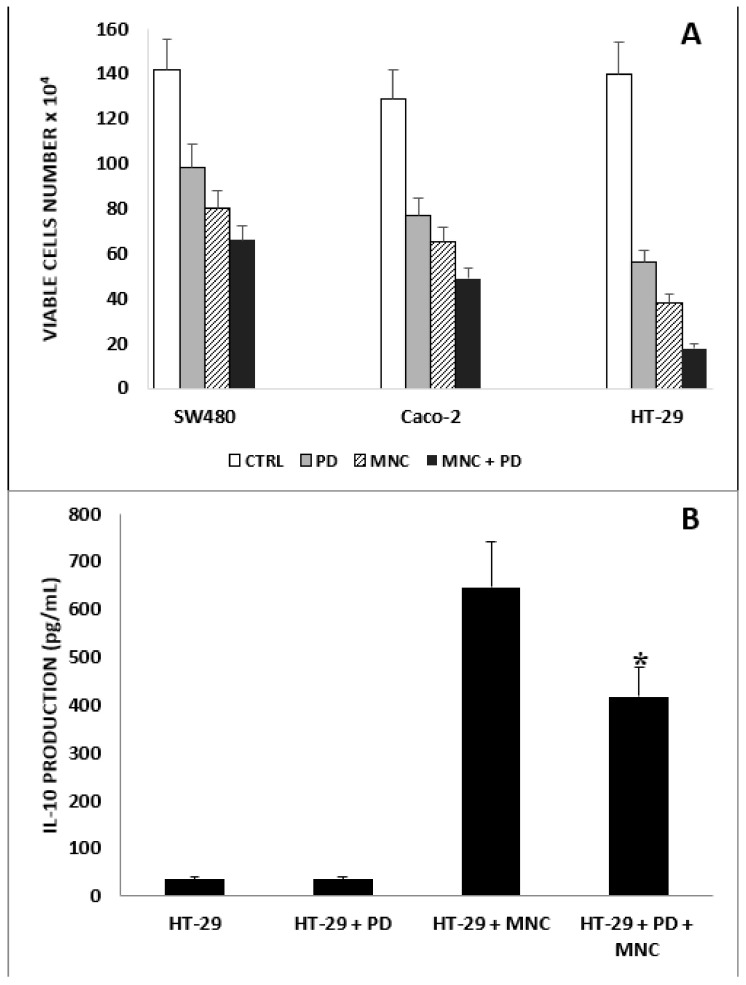
Effect of PD on CRC/MNC coculture and IL-10 production. (**A**). Effect of PD (5 μg/mL) on three human CRC cell lines cocultured with MNCs at a CRC/MNC ratio of 1:10 for 72 h. Results are expressed in terms of the geometric mean (each group in triplicate) of viable cell counts. Bars represent the SE of the geometric mean. In all cases, all single treatments produced a significant inhibition (*p* < 0.05) of cell growth with respect to untreated controls. *p* < 0.01 in HT-29/MNCs treated with PD (5 μg/mL) versus HT-29/MNCs. (**B**). Effect of PD (5 µg/mL) on IL-10 production in HT-29/MNC coculture described in (A). Results are expressed in terms of IL-10 production (pg/mL). The IL-10 production was detected by ELISA. Data are expressed as the mean of three independent experiments. Bars represent the SE of the mean. * *p* < 0.01, HT-29 + PD + MNCs vs. HT-29 + MNCs.

A model of in vitro short-term (i.e., 72 h) interaction between non-pretreated immunocompetent human peripheral blood mononuclear cells (MNCs) and CRC cells (CRC/MNC ratio of 1:10) was used in several experiments illustrated later. At the beginning, we tested the possible influence of PD in this coculture model. The results (Figure 2A) confirmed that a low concentration of PD alone significantly reduces CRC cell growth. In turn, the presence of MNCs alone in the coculture produced a significant decline in CRC cell proliferation. A further decrease in the number of viable CRC cells was observed when PD was added to the CRC/MNC cocultures. In particular, a strong antiproliferative effect (reaching approximately 80% value) was observed in HT-29/MNC cocultures treated with PD.

Additional experiments were performed to exclude a cytotoxic effect of PD on MNCs. The effect of PD at a concentration of 5 µg/mL for 24, 48, and 72 h was evaluated in terms of cell viability and apoptosis induction. The results (data not shown) showed that cell death and apoptosis do not exceed 8% at any time of MNC exposure to drug treatment. Similar results were obtained when MNCs were pre-stimulated with LPS (1 µg/mL for 4 h).

Supernatants (SN) of HT-29/MNC (1:10) cocultures treated or not treated with PD (5 µg/mL) were collected for IL-10 detection after 72 h of culture. The cytokine was measured in undiluted cell culture SN and tested in quadruplicate.

The results illustrated in Figure 2B show that IL-10 was not detectable in the SN collected from wells containing HT-29 alone or in the presence of PD. However, significant levels of IL-10 were found when tumor cells were cocultured with MNCs. In these conditions, the treatment with PD substantially downregulated (i.e., by 40%) the production of IL-10.

### 2.2. Effect of PD and LPS on HT-29 Cell Migration

**Figure 3:**
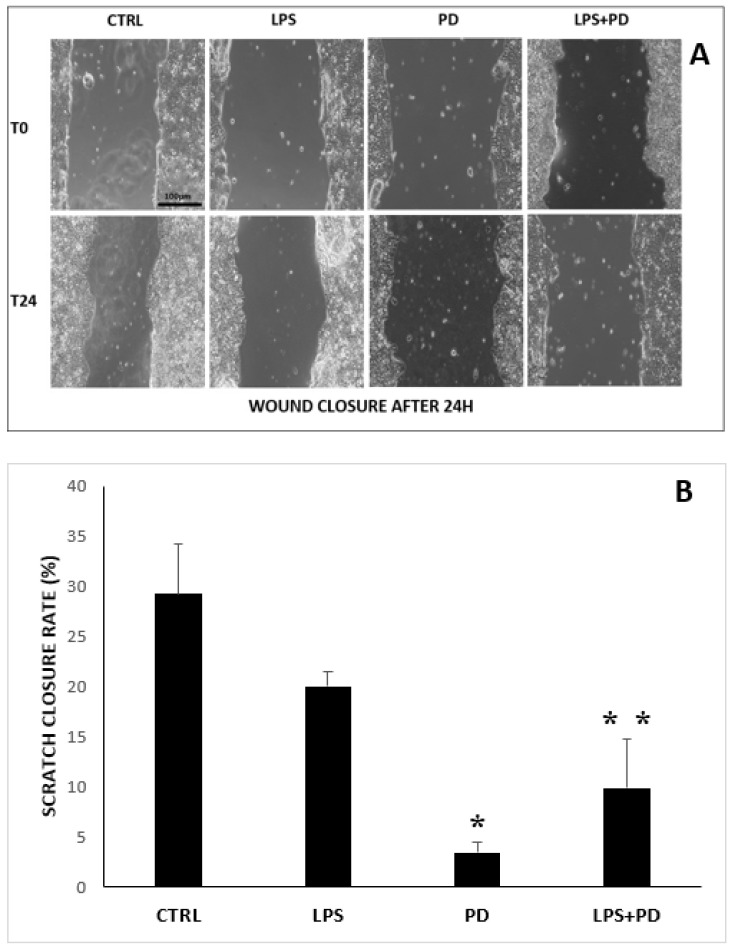
Effects of PD on migration of HT-29 cells. Comparison of migratory activity of control cells and LPS/PD-stimulated HT-29 cells. (**A**) Culture of HT-29 untreated control cells, cells treated with 5 µg/mL PD, or cells treated with 1 µg/mL PD+LPS at time 0 (upper plates, 0 h) or after 24 h (lower plates, 24 h). Photographs were taken at x10 magnification using a digital camera under an inverted phase-contrast microscope (Olympus). (**B**) The closure of the scratch was quantified by measuring the difference between the wound width at T0 and T24, using ImageJ software (downloaded from NIH web site: http://rsbweb.nih.gov/ij/); The scratch closure rate (SCR) was calculated using the following formula: SCR = [(At0 − At24)At0] × 100. Results are expressed in terms of the means of three independent experiments ±SD. * *p* < 0.01, treated with PD (5 µg/mL) vs. HT-29 controls. ** *p* < 0.01, HT-29 cells treated with LPS (1 µg/mL) and PD (5 µg/mL) vs. HT-29 cells treated with LPS.

We evaluated the effect of PD and LPS on HT-29 cell migration in vitro by a scratch wound-healing assay. Cell migration ability was calculated as the area covered by adherent cells after 24 h of culture, as illustrated in Figure 3A,B.

HT-29 cells were treated with LPS alone (1 µg/mL for 24 h) in order to evaluate the effect of LPS (normally present in the colon microenvironment) on cancer cell migration. The results showed that LPS induces low wound closure inhibition with respect to untreated controls, i.e., 20% vs. 29%. When HT-29 cells were exposed to PD (5 µg/mL for 24 h), the wound closure rate notably decreased with respect to untreated controls, i.e., 3% vs. 29%. Moreover, PD exerted its inhibitory effect even in the presence of LPS, i.e., 11% vs. 20%.

We also examined the effect of 24 h treatment with LPS (1 µg/mL) on the proliferative activity of HT-29 cells. Untreated or LPS-treated HT-29 cells were tested using colorimetric MTT assay. The results showed that LPS does not have any significant influence on HT-29 cell proliferation (data not shown).

### 2.3. Anti-Inflammatory Activity of PD on HT-29 Cells Treated with LPS

**Figure 4:**
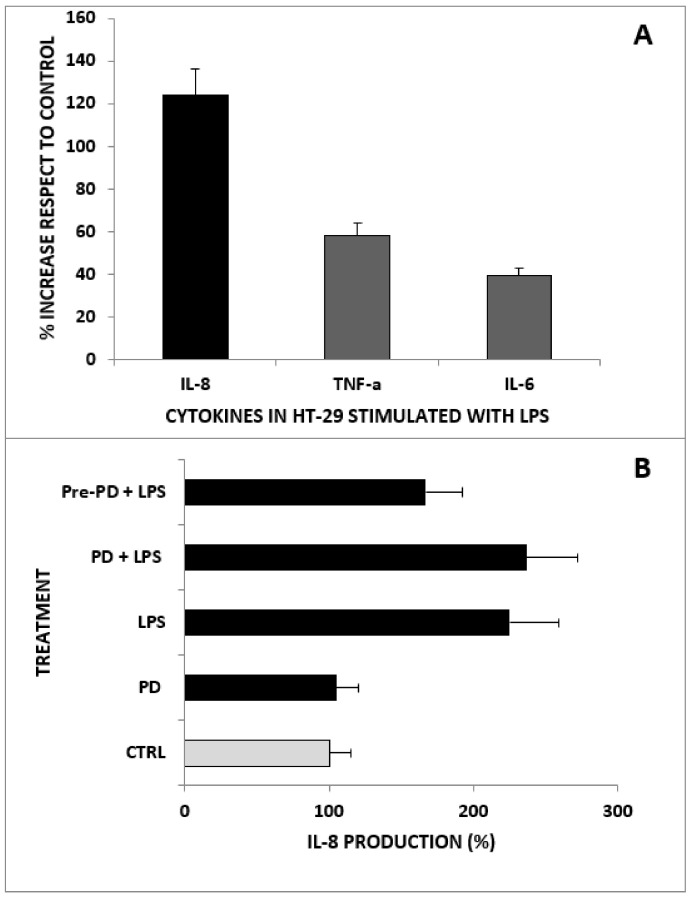
Cytokine production by LPS-stimulated HT29 cells. (**A**) Proinflammatory cytokine production (IL-8, IL-6, and TNF-α) by HT-29 cells treated with LPS (1 µg/mL) for 16 h. Results are expressed in terms of the percentage increase in cytokine production with respect to untreated HT-29 (controls). Data are expressed as the mean of three independent experiments. Bars represent the SE of the percentages. (**B**) IL-8 production by HT-29 cells treated with LPS and/or PD. The treatments were performed with LPS (1 µg/mL), PD (5 µg/mL), PD (5 µg/mL) before LPS (1 µg/mL), or PD (5 µg/mL) plus LPS (1 µg/mL). Results are expressed in terms of the percentage of cytokine production with respect to untreated HT-29 controls. Data are expressed as the mean of three independent experiments. Bars represent the SE of the percentages. Pre-PD + LPS is significantly (*p* < 0.05) lower than PD + LPS.

The anti-inflammatory activity of PD was assessed in vitro in the HT-29 cell line by measuring the concentration of proinflammatory cytokines, such as IL-8, IL-6, and TNF-α, released by LPS-treated malignant cells. It was found that cancer cells treated with LPS (1 µg/mL) for 16 h displayed secretion levels of IL-8, IL-6, and TNF-α significantly higher than those of untreated cells, as illustrated in Figure 4A. Pretreatment of HT-29 cells with PD (5 µg/mL) for 24 h before LPS stimulation significantly reduced (225% vs. 167%) the increase in LPS-induced IL-8 production (Figure 4B). However, when PD was used in combination with LPS, no significant effect was observed.

As previously described (Figure 1A), PD showed no cytotoxic effects on the cell viability of HT-29 cells after 24 h.

Given that HT-29 cells can express all the necessary components of LPS-induced TLR-4 signaling [13], we hypothesized that pretreatment with PD could downregulate TLR-4 expression, reducing the inflammatory response to the LPS stimulus. The results obtained by Western blot analysis, confirmed by three independent experiments (data not shown), did not support this hypothesis. Actually, HT-29 cells express TLR-4, but in our experimental conditions, this was not modulated by PD.

### 2.4. The Effect of PD on CAM Expression of HUVECs

We examined the effect of PD on cell adhesion molecules (CAMs) by which tumor cells bind to endothelial cells. The CAMs were evaluated on HUVECs that express many important endothelial markers and responses to various physiological and pathophysiological stimuli.

**Figure 5:**
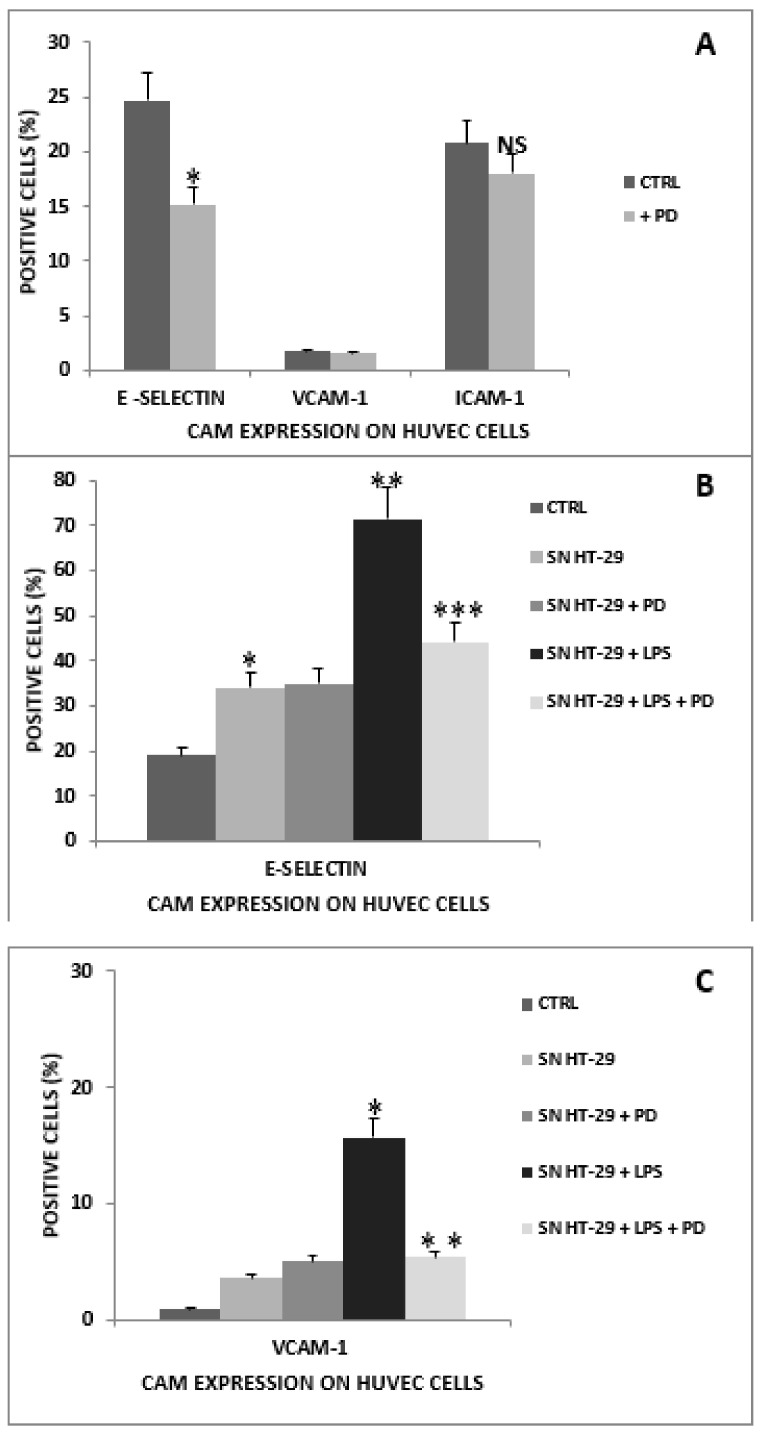
The expression of CAMs on HUVECs. (**A**) Effect of PD (5 µg/mL) on CAMs of HUVECs. Results are expressed as the mean of the percentage of E-selectin-, VCAM-1-, and ICAM-1-positive cells evaluated by flow cytometry. The experiments were performed in triplicate. Bars represent the standard errors of the mean. * *p* < 0.01. NS (not significant) (**B**) Endothelial E-selectin expression in HUVECs. HUVECs were untreated or exposed to SN obtained from HT-29 cells: untreated (SNHT-29), LPS-stimulated (1 µg/mL; SN HT-29 + LPS), treated with PD (5 µg/mL; SN HT-29 + PD), and treated with PD (5 µg/mL) and LPS (1 µg/mL; SN HT-29 + LPS + PD). Results are expressed as the percentage of cells positive for E-selectin evaluated by flow cytometry. The experiments were performed in triplicate. Bars represent the mean of percentages. * *p* < 0.01, HUVECs treated with SNHT-29 cells vs. HUVEC controls. ** *p* < 0.01, HUVECs treated with SN of HT-29 cells exposed to LPS vs. HUVECs cultivated with SN of non-treated HT-29 cells. *** *p* < 0.01, HUVECs treated with SN of HT-29 cells exposed to LPS + PD vs. HUVECs exposed to SN of LPS-treated HT-29 cells. (**C**) Endothelial VCAM-1 expression in HUVECs. HUVECs were untreated or exposed to SN obtained from HT-29 cells: untreated (SN HT-29), LPS-stimulated (1 µg/mL; SN HT-29 + LPS), treated with PD (5 µg/mL; SN HT-29 + PD), and treated with PD (5 µg/mL) and LPS (1 µg/mL; SN HT-29 + LPS + PD). Results are expressed as the percentage of cells positive for VCAM-1 evaluated by flow cytometry. The experiments were performed in triplicate. Bars represent the standard errors of the mean. * *p* < 0.01, HUVECs treated with SN of HT-29 cells exposed to LPS vs. HUVECs cultivated with SN of non-treated HT-29 cells. ** *p* < 0.01, HUVECs treated with SN of HT-29 cells exposed to LPS + PD vs. HUVECs exposed to SN of LPS-treated HT-29 cells.

The expression of CAMs on HUVECs was assessed after treatment with PD (5 µg/mL) for 24 h. The results, illustrated in Figure 5A, showed that PD is able to down significantly regulate E-selectin, whereas the percentage of cells positive for VCAM-1 and ICAM-1 remained substantially unchanged.

As shown in Figure 5B, in HUVECs, the percentage of cells positive for E-selectin expression significantly increased after exposure to the SN of HT-29 cells. Moreover, the increase in E-selectin expression was significantly higher when HUVECs were treated with SN obtained from LPS-treated HT-29 cells. The treatment of HUVECs with the SN of HT-29 was not followed by a significant increase in the percentage of cells positive for VCAM-1. However, when HUVECs were exposed to SN obtained from LPS-treated HT-29 cells, a significant increase in VCAM-1 was detected, as shown in Figure 5C.

The effect of PD (5 µg/mL) on CAM expression of HUVECs cultured in the presence of the SN of LPS-treated (1 µg/mL) HT-29 cells was also investigated. The results showed that PD is able to significantly reduce the percentage of cells positive for E-selectin expression (30–40%) and the percentage of cells positive for VCAM-1 expression (about 30%), as illustrated in Figure 5B,C, respectively. In the same experimental conditions, the expression of ICAM-1 was not significantly modified (data not shown).

Additional experiments were performed to evaluate the effect of PD (5 μg/mL) for 24 h on HUVEC viability. The results indicated that PD does not have any significant effect on HUVEC number and viability (data not shown).

## 3. Discussion

Several years of preclinical and clinical investigations have shown that the tumor microenvironment (TME) is particularly complex and plays a critical role in tumor progression/regression and metastatic diffusion. Inflammation, which is typically and constantly associated with the TME, is driven by proinflammatory mediators that are secreted by a variety of cells present in the TME, i.e., tumor cells, tumor-infiltrating lymphocytes, macrophages, and myeloid and dendritic cells [33].

The possibility to modulate the inflammation present in the TME by natural substances has elicited considerable interest in cancer research.

RES and PD are natural antioxidant and anti-inflammatory agents with low toxic effects against normal cells, including colon epithelial cells [34]. These two compounds have well-known anti-tumor efficacy by inducing tumor cell apoptosis and cell cycle arrest [24,25,26,27,28,29,30,31,32]. Conversely, the effect of these compounds on the TME in terms of the cytokine network, cancer progression, and metastasis has not been fully investigated.

The aim of this study was to confirm the ability of PD to control colon cancer cell growth in terms of proliferation blockade and apoptosis induction and to examine its effect on the TME using an in vitro model.

PD is the glycosylated molecule of RES [31] and was adopted for our investigation since glycosylation enhances the solubility of this natural molecule and provides better bioavailability in vivo with respect to RES.

The results showed that PD possesses in vitro anti-tumor effects against all colon cancer cells examined. In particular, PD shows significant antiproliferative effects on HT-29 cells in a time- and concentration-dependent manner and proapoptotic effects at concentrations that are lower than those required for RES, as previously described [26]. Moreover, the results obtained treating the Caco-2 cell line with low concentrations of PD are in line with those obtained by De Maria et al. [28].

In this investigation, we used a simple model with the intent to reproduce, at least in part, the pathological conditions of the colon cancer TME, including the presence of MNCs and cytokine-dependent inflammation and immunodepression.

The results showed that the in vitro growth of CRC cells is reduced by MNCs alone and that this effect is marked when a low dose of PD is added to the MNCs. This antiproliferative effect is more evident in HT-29 cells.

It is reasonable to hypothesize that PD could improve natural cell-mediated immunity, as previously suggested for RES by us and other authors [27,35,36].

It is known that TME cytokines are able to either inhibit or promote cancer progression. In particular, IL-10 is one of the Th2-associated cytokines that induces an immunosuppressive microenvironment [37]. IL-10 inhibits the anti-tumor immune reaction, suppressing cytotoxic T cell functions by immune-suppressive (CD4+CD25+) Treg cells [38]. We focused on this cytokine since IL-10 is frequently upregulated in various types of cancer. Actually, a strong correlation was found between serum IL-10 levels, disease progression, and metastases [39]. In the HT-29/MNC cocultures, PD significantly reduced the level of this immunosuppressive cytokine. To exclude the hypothesis that the reduction in IL-10 levels could be the result of drug-induced downregulation of the MNC number, we evaluated the cell count and apoptosis of MNCs treated with 5 µg/mL of PD and found that the agent is not toxic in these experimental conditions.

Moreover, it is well known that IL-10 and LPS-stimulated macrophages modulate several biochemical pathways in the TME of colorectal cancer. Both LPS- and IL-10-stimulated macrophages induce the phosphorylation of EGFR, Akt, and ERK1/2 in cancer cells [40]. It is possible that PD-induced reduction in IL-10 in the tumor microenvironment could affect indirectly EGFR, thus preventing downstream activation of Akt and ERK1/2 kinases in malignant cells. In addition, it has been found that RES directly decreases EGFR-mediated signaling [41], so it is reasonable to hypothesize that PD could inhibit cancer cell growth either directly or indirectly by reducing immunological escape mechanisms.

It is known that CRC cells are frequently surrounded by inflammatory cells and intestinal microbiota, including *Escherichia coli* that typically produces copious amounts of LPS. Therefore, LPS could promote colon cancer metastasis by enhancing tumor cell adhesion, intravasation, and extravasation [42].

The presence of LPS in an inflamed microenvironment could induce an increase in the proliferation and migration of cancer cells [43], although this effect is still debated [44,45]. To evaluate the effect of PD on cancer cell migration, we treated HT-29 cells with LPS and/or PD for 24 h and evaluated the directional cell migration in vitro. The results showed that PD significantly inhibits HT-29 cell migration, although the combined treatment with LPS reduces this effect. Additional experiments were performed to evaluate the effect of LPS on HT-29 cell growth to exclude that the decrease in LPS-treated HT-29 cell migration is associated with an antiproliferative effect. The results by MTT assay pointed out that LPS does not affect HT-29 cell growth (data not shown).

In the colon microenvironment, inflammation is a crucial response against various stimuli, such as LPS [46]. High expression of inflammatory cytokines, including IL-8, TNF-α, and IL-6, may play a central role in oxidative-stress-induced inflammation in the TME.

Our results, in line with the findings described by other authors, showed that HT-29 cells, subjected to LPS stimuli, release in vitro inflammatory cytokines, including IL-8, TNF-α, and IL-6 [47,48]. The anti-inflammatory activity of PD was evaluated by pretreating or treating HT-29 cells in combination with LPS. The results showed that PD significantly reduces the secretion of IL-8 in LPS-stimulated HT-29 cells only when the cells are pretreated with PD before LPS stimulation.

TLR-4 represents a key receptor responsible for inflammatory reactions upon infectious and noninfectious stimuli [49]. Our results indicated that TLR-4 expressed by HT-29 cells is not modulated by PD, thus suggesting that the inhibition of the inflammatory response by PD involves downstream signaling of other components, e.g., NF-κB, that is downregulated by RES [50].

In solid tumors, the multi-stage metastatic process is facilitated by the adhesion of cancer cells to the endothelium. It is known that CAMs, including selectins, integrins, and immunoglobulin superfamily (IgSF) proteins, play an essential role in metastasis. In fact, during extravasation, tumor cells penetrate the vascular compartment and adhere to the endothelium [18].

It is well known that flavonoids can control the expression of endothelial adhesion molecules [51], and recent studies suggest that RES modulates the inflammatory profiles of immune and endothelial cells [52,53]. In addition, a study examined the inhibitory effect of PD on inflammation-induced cell-to-cell adhesion in the endothelial cell and monocyte system. This study attributed mainly the inhibitory effect of PD on NF-κB pathway activation [54]. To the best of our knowledge, there are no studies looking at the inhibitory effects of PD on the vascular invasion of colon tumor cells stimulated by the presence of LPS.

The effects of cytokines and proinflammatory mediators on adhesion molecule expression in the LPS-stimulated microenvironment are not completely explored. In a previous study, Simiantonaki et al. [19] demonstrated that the SN of LPS-stimulated colon cancer cells upregulate adhesion molecules on endothelial cells. Then, the aim of this study was also to test whether PD can modulate the expression of adhesion molecules, such as ICAM-1, VCAM-1, and E-selectin, induced in HUVECs by the SN of LPS-stimulated HT-29 cancer cells. Therefore, HT-29 cells were exposed to LPS, followed by treatment with PD. In line with previous studies [19], we found that the SN of LPS-stimulated malignant cells produce a marked increase in the expression of ICAM-1 VCAM-1, and E-selectin on HUVECs. This effect was more remarkable with respect to that seen in HUVECs exposed to the SN of non-stimulated cancer cells. Moreover, we demonstrated that PD significantly reduces the percentage of cells positive for the expression of E-selectin and VCAM-1 in HUVECs subjected to the SN of LPS-stimulated HT-29 cells. These results are in line with previous findings that showed the inhibitory effect of PD on E-selectin and VCAM-1 expression on HUVECs treated directly with LPS [51].

In addition, we evaluated the effect of PD on HUVECs. The results showed that PD significantly reduces the expression of E-selectin, whereas it does not significantly modify the number of cells positive for ICAM-1 and VCAM-1 expression.

Both PD and RES are potent activators of sirtuin 1 (SIRT-1) that plays a vital role in protecting the small intestine, reduces microvascular inflammation, and limits systemic injury due to sepsis [55]. Moreover, Wang et al. [56]. suggested that RES reduces the expression of E-selectin/ICAM-1 by the induction of SIRT-1. Taking into account these previous results, we could hypothesize that PD reduces CAM expression through a similar mechanism.

The preliminary results illustrated in this report show that the inflammation and metastatic potential associated with the TME could be counteracted by PD and suggest a rationale for the use of PD in the clinical management of colorectal cancer.

## 4. Materials and Methods

### 4.1. Cell Lines and Cell Culture

The human umbilical vascular endothelial cell (HUVEC) line (JG-C2517A, single donor) and its medium (EGM^®^-2; endothelial growth medium with BulletKit^®^ (CC-3162)) were purchased from Lonza (Walkersville, MD, USA). HUVECs were cultured in EGM-2 supplemented with 10% heat-inactivated fetal calf serum (FCS; Hyclone Laboratories, Logan, UT, USA), endothelial growth supplement (CC-4113), hEGF, hydrocortisone, 5% penicillin/streptomycin (CC-4381), and 25 μg/mL of heparin (CC-4936) and maintained in a 5% CO_2_ humidified atmosphere at 37 °C. All experiments used cells collected after two or three passages in tissue culture.

HT-29, SW480, and Caco-2 human colon cancer cells were obtained from the American Type Culture Collection (Manassas, VA, USA). Adherent HT-29 cells were cultured in RPMI-1640 (Hyclone Europe, Cramlington, UK) supplemented with 10% FCS, 2 mM L-glutamine, and 1% streptomycin/penicillin and referred to as the complete medium (CM). The SW480 and Caco-2 cell lines were cultivated with Dulbecco’s Modified Eagle Medium (DMEM) containing 10% fetal bovine serum (FBS) and 1% streptomycin/penicillin and referred to as CM. All of them were kept at 37 °C with 5% CO_2_. All cells were subcultured following detachment from the plastic surface using trypsin/EDTA solution.

### 4.2. Reagents

Natural polydatin (3,5,4′-trihydroxystilbene 3-O-beta-D-glucopyranosid PD) was extracted and kindly supplied by Dr. Fulvio Mattivi (Fondazione Edmund Mach, San Michele All’Aadige, Trento, Italy). The purity of the compound, tested according to the patented method (WO2001091763A3 Extracts from spermatophyte plants with antitumor activity by F. Mattivi, et al., 2002) reported in, was higher than 99%. The drug was dissolved in RPMI-1640 (GIBCO Laboratories, Grand Island, NY, USA) at 100 mM. Lipopolysaccharide (LPS) was obtained from Sigma Chemical Co. (St. Louis, MO, USA). All stock solutions were stored at −80 °C and diluted in the culture medium just prior to use.

Human mononuclear cells (MNCs) were obtained from voluntary healthy donors using LYMPHOLYTE^®^-H Cell Separation Media (catalog no. CL5020; CEDARLANE^®^, Burlington, ON, Canada).

Cytokines: The amount of human IL-10 was determined using the Human IL-10 Quantikine ELISA Kit D1000B-R&D Systems. The amount of human IL-8, IL-6, and TNF-α was determined using, respectively, the Human IL-8 ELISA Kit II (catalog no. 550999; BD OptEIA™), IL-6 ELISA Kit II (catalog no. 550799; BD OptEIA™), and Human TNF-α ELISA Set (catalog no. 555212).

Antibodies: against E-selectin, PE anti-human CD62E; against VCAM-1, PE mouse anti-human CD106; and against ICAM-1, PE mouse anti-human CD54. All antibodies and isotype control mouse IgG2a were purchased from BD Pharmingen™.

### 4.3. Determination of Cell Growth and Apoptosis Induction

HT-29 cells were seeded in 24-well tissue culture plates (Falcon) at a concentration of 5 × 10^4^ cells/mL and allowed to adhere overnight. Cells were incubated with graded concentrations of PD (5, 10, 20, and 40 μg/mL) or the medium alone as a control. The plates were incubated at 37 °C in a 5% CO_2_ humidified atmosphere for 24, 48, or 72 h. Cell growth and viability were evaluated every 24 h. Trypsinized cells were manually counted using a hemocytometer, and cell viability was determined using trypan blue dye exclusion assay. All determinations were made in triplicate, and the results were expressed in terms of the geometric mean ± standard error.

To evaluate apoptosis in HT-29 cells or in MNCs treated with PD, adherent and floating cells were collected from each sample treated with graded concentrations of PD (i.e., 5, 10, 20, and 40 μg/mL) for 48 h. The cells were then fixed (acetone/methanol 1:4 in PBS 50%), washed twice with PBS, and stained with 50 µg/mL of propidium iodide (PI) in the presence of 100 KU/mL of RNase A. Cellular fluorescence was measured with a FACSscan flow cytometer (Becton Dickinson, Franklin Lakes, NJ, USA) using the argon+ ion laser emitting at 488 nm. Apoptotic cells are represented by a broad hypodiploid peak (cells with a fractional DNA content), which is easily distinguishable from the narrow peak of cells with diploid DNA content in the red fluorescence channel. The fraction of apoptotic cells was therefore calculated by integrating the pre-G1 peak. This fraction is representative of cells with decreased staining for PI as an indicator for DNA fragmentation associated with apoptotic cell death.

### 4.4. Wound-Healing Assay

To further assess the effects of PD on directional cell migration in vitro, a wound-healing assay was performed [44]. Briefly, 3 × 10^5^ cells were cultured in each well of a 6-well plate at 37 °C for 24 h, and then a straight scratch was made using a pipette tip on the confluent cell monolayer. Fresh CM was added to remove the floating cells, and the remaining cells were imaged immediately (i.e., time 0 h) using an inverted microscope. Images were recorded using digital photography. The culture medium was removed and replaced with fresh medium containing PD alone (5 µg/mL) and/or LPS (1 µg/mL) for 24 h. Following incubation at 37 °C for 24 h (i.e., time 24 h), images were captured and the closure of the scratch was quantified by measuring the difference between the wound width at T0 and T24 using ImageJ software (downloaded from NIH web site: (http://rsbweb.nih.gov/ij/); The scratch closure rate (SCR) was calculated using the following formula: SCR = [(At0 − At24)At0] × 100 [57].

### 4.5. CRC and MNC Coculture: Cell Growth and IL-10 Evaluation in Supernatants

Human MNCs obtained from the peripheral blood of healthy donors were resuspended in CM. CRC cells at a concentration of 10^5^ cells/mL were allowed to adhere in 24-well tissue culture plates (Falcon) at 37 °C in a 5% CO_2_ humidified atmosphere for 16 h.

Untreated MNCs containing monocytes/macrophages suspended at a concentration of 10^6^ cells/mL were added to CRC cells in order to obtain a 10:1 mixture of MNCs/CRCs. The cocultures were incubated in the absence (control) or in the presence of PD (final concentration of 5 µg/mL) in a total volume of 2 mL for 72 h. Trypsinized cells were manually counted using a hemocytometer, and cell viability was determined by trypan blue dye exclusion assay. All determinations were made in triplicate, and the results were expressed in terms of the geometric mean ± standard error.

The SN of HT-29/MNC cocultures treated or not treated with PD were collected for IL-10 determination after 72 h of culture. Each culture was set in triplicate. The test was carried out using the Human IL-10 Quantikine ELISA Kit. All determinations were made in quadruplicate, and the results were expressed in terms of the geometric mean ± standard error.

### 4.6. Cell Treatments: CAM Evaluation and Inflammatory Cytokine Production

To evaluate CAM expression on human umbilical vascular endothelial cells (HUVECs), HT-29 cells were treated with PD (5 µg/mL) for 4 h. Thereafter, the cells were treated with LPS (1 µg/mL) for further 4 h. At the end of treatments, the cells were washed extensively and incubated overnight in new CM. Afterward, the SN were harvested and added to confluent HUVEC cultures for 4 h. After treatment, HUVEC monolayer samples were labeled with mouse monoclonal antibodies against adhesion molecules (E-selectin, PE anti-human CD62E; VCAM-1, PE mouse anti-human CD106; and ICAM-1, PE mouse anti-human CD54) at 4 °C for 30 min. Subsequently, cells were washed with PBS and then fixed with 0.5% paraformaldehyde (PFA) in PBS at 4 °C for 2 min. Finally, HUVEC monolayers were treated with trypsin/EDTA at room temperature for 5 min. The cells were carefully scraped, washed with PBS, and suspended in PBS for flow cytometry. A FACScan Flow Cytometer (Becton & Dickinson USA) was used to analyze samples. In total, 10,000 events were analyzed for each test, and signals from all parameters were captured as list mode data and analyzed by FACScan research software. All experiments were performed in triplicate.

To investigate the inflammatory effect of LPS on HT-29 cells, the levels of three proinflammatory cytokines, namely IL-6, IL-8, and TNF-α, were evaluated in the SN of cultured cells either untreated or pretreated with LPS (1 µg/mL) overnight. Using this experimental model, additional groups were treated with PD (5 µg/mL) 24 h before exposure to LPS or during LPS incubation. In all cases, cytokine levels have been tested by ELISA assays, as previously described.

### 4.7. Statistical Analysis

The results were expressed as the mean ± standard error (SE) or standard deviation (SD) of the mean from three independent experiments (*n* = 3). Data were subjected to one-way analysis of variance (ANOVA, San Francisco, CA, USA), and the statistical differences between samples were compared using Dunnett’s test. All statistical analyses were carried out with GraphPad Prism 9.0.2 (GraphPad Software, San Diego, CA, USA). A *p*-value of <0.05 was considered statistically significant.

## Data Availability

Not applicable.
